# Therapeutic potential of cannabis for surgical wound healing in rats

**DOI:** 10.17221/21/2024-VETMED

**Published:** 2024-08-16

**Authors:** Gokhan Dogukan Akarsu, Rukiye Hobek Akarsu

**Affiliations:** ^1^Laboratory for Advanced Genomics, Division of Molecular Medicine, Institute of Ruder Boskovic, Zagreb, Croatia; ^2^Pharmacy Services, Vocational School of Health Services, Yozgat Bozok University, Yozgat, Turkiye; ^3^Department of Nursing, Faculty of Health Services, Yozgat Bozok University, Yozgat, Turkiye

**Keywords:** cannabinoids, cannabis sativa, essential oil, hemp, treatment, wounds and injury

## Abstract

This study was conducted to evaluate the wound-healing activities of a *Cannabis sativa* L. plant extract and cannabidiol on incision wounds. An incision was created and sutured in rats under anaesthesia. Routine wound care procedures were applied for 10 days, followed by histological wound examinations. The cellular bioactivities of the hemp extract and CBD were assessed for MCP-1, EGF, BFGF, IL-8, and COL-1 using ELISA on the rat skin wound healing activity. A one-way ANOVA was used for the data analysis. The EGF values in the plasma were similar in the povidone-iodine, hemp seed oil, and hemp essential oil groups (*P* > 0.05). However, the EGF levels were lower in the CBD group compared to the other groups (*P* < 0.001, *P* < 0.005). The MCP-1 values in the hemp seed oil, hemp essential oil, and CBD were similar (*P* > 0.05), whereas povidone iodine exhibited lower MCP-1 levels compared to the other groups (*P <* 0.001, *P* < 0.005). It was determined that the plasma BFGF, IL-8, and COL 1 values of the groups were similar (*P* > 0.05). To our knowledge, this study is the first to evaluate the effects of CBD, seed oil, and hemp leaf extract on incision wound healing. It demonstrates that hemp extract holds greater potential benefits for wound healing compared to CBD.

Wound healing is a complex biological process divided into four overlapping stages: haemostasis, inflammation, proliferation, and remodelling (Abbas et al. 2019). Depending on the depth and severity of the wound’s thickness, wound healing takes several months (6–9 weeks). Due to intense inflammation and a higher rate of infection (bacterial proliferation), non-healing wounds pose a challenging approach for wound care specialists worldwide, especially in an ageing population, and in patients suffering from diabetes and obesity. Classical or traditional wound therapy usually uses gauze dressings, which do not have a therapeutic effect. They are inexpensive, but the outcome of the wound treatment with traditional dressings is often uncertain, and the process could be economically inefficient. Non-toxic and non-allergenic dressing materials are necessary, which allow gas exchange, reduce inflammation by creating a barrier against microbial agents, and support biological healing processes by preventing infection (Xu et al. 2015). Various wound dressing products have been developed using synthetic polymers to expedite wound healing (Hou et al. 2020). Synthetic polymers can potentially cause skin damage or lead to infection due to their structures. However, there is insufficient evidence to demonstrate the superiority of wound dressing products over synthetic polymers or traditional dressing methods (Mirasoglu 2015). As a result, research on naturally derived products with antibacterial and pro-collagen synthesis properties is continuing to advance to achieve optimal outcomes in wound healing. Numerous studies suggest that cannabinoids, due to their anti-inflammatory and antioxidant effects, might offer a novel approach to wound healing by alleviating inflammation and supporting the healing process (Thapa et al. 2020; Kibret et al. 2022; Kongkadee et al. 2022; Spinella et al. 2023).

Cannabinoids, biologically active compounds derived from *Cannabis sativa* (phytocannabinoids), have garnered attention for their anti-inflammatory and antioxidant effects, suggesting a novel approach to wound healing by alleviating inflammation and supporting the healing process (Passani et al. 2020). Despite the ancient recognition of the biological and pharmacological properties of *Cannabis sativa* in popular medicine, its pharmacological use has been restricted due to concerns about misuse related to the presence of tetrahydrocannabinol THC (Andre et al. 2016).

In modern times, the sativa subspecies is legally cultivated in many countries, not only for its seeds, an excellent source of lipids, proteins, carbohydrates, minerals, and vitamins, but also for the production of “Cannabidiol = CBD” (non-psychoactive cannabinoid), which is of significant importance (Ferrini et al. 2021). The existing literature primarily focuses on CBD, overlooking the biologically valuable molecules present in both the seeds and leaves of *Cannabis sativa*, which hold high nutritional value (Callaway 2004; Wang et al. 2008; Da Porto et al. 2012; Teh and Birch 2013; Pojic et al. 2014; Liang et al. 2015; Schluttenhofer and Yuan 2017; Mikulec et al. 2019; Rapa et al. 2019).

Biochemical markers used to determine wound healing:

- Epidermal growth factor (EGF): It plays an important role in the regulation of cell growth, proliferation and differentiation (Gurtner and Wong 2006).
- Monocyte chemoattractant protein-1 (MCP-1/MCAF): It has been shown that the MCP-1 protein increases in the wound area. It has been reported that it regulates keratinocyte migration (Gurtner and Wong 2006).
- Basic fibroblast growth factors (BFGFs): By stimulating angiogenesis and the proliferation of fibroblasts, they form the granulation tissue that fills the wound cavity in the early phase of wound healing (Barrientos et al. 2008).
- Interleukin-8 (IL-8): The IL-8 level has been shown to increase in the dermis of acute surgical wounds (Metcalfe and Ferguson 2007; Raja et al. 2007).
- Collagen-1 (COL-1): COL-1 has a central role as the main structural component of the dermis in the remodelling of cutaneous tissue defects (Metcalfe and Ferguson 2007; Raja et al. 2007).

However, there is no study in the literature evaluating the superiority of both the seed oil and leaf extract over pure CBD or over routinely used dressing products. Therefore, the aim of this study is to evaluate the effectiveness of hemp essential oils, hemp seeds, and CBD against 10% povidone-iodine, routinely used in surgical operations for abdominal sutured incisions in rats.

## MATERIAL AND METHODS

### Ethical statement

The animal study protocol was approved by the Erciyes University Animal Experiments Ethics Committee (04/21/79).

### Chemicals

“Dimethyl sulfoxide = DMSO” (purity > 99.5%) and ethanol (purity > 99.5%) from Sigma Aldrich (Massachusetts, USA), CBD (purity > 95%) from Yozgat Bozok University Hemp Research Institute (Yozgat, Türkiye), Primavilin from VILSAN Veterinary Medicines Trade and Industry Company (Ankara, Türkiye), Ketolin from Pİ Farma İlaç Industry and Trade Company (Ankara, Türkiye), Xylazine (Bioveta PLC, Ivanovice na Hane, Czech Republic), Ketamine (Richter Pharma AG, Wels, Austria) were obtained and 10% povidone iodine was purchased from Alfa Medical Health Products Company (Istanbul, Türkiye).

### Study design

#### *CANNABIS SATIVA* PROCUREMENT

The hemp was obtained from Yozgat Bozok University, which has a “Hemp Research Institute” and was authorised with special permission. All phases of the research were carried out within legal responsibilities and limitations.

#### PREPARATION OF THE CBD

The CBD was obtained from the Hemp Research Institute of Yozgat Bozok University. The CBD prepared by the institute is 98% pure. CBD was stored at 4 °C until the CBD solution was prepared. To prepare the solution, it was brought to room temperature and 96% ethanol was added. The resulting mixture was vortexed to obtain a solution containing 15% CBD by volume. The prepared solution was transferred to a spray can and wrapped with aluminium foil to prevent exposure to light.

#### PREPARATION OF THE SEED OIL

Hemp seed oil was obtained from hemp seeds by cold pressing. The obtained oil was stored at 4 °C until required during the experiment. It was wrapped in aluminium foil to avoid exposure to light.

#### PREPARATION OF THE LEAF EXTRACT

The process steps for obtaining the essential oil of hemp are as follows: The green parts of the hemp were harvested in season. Since drying in the oven may damage the essential oils, it was allowed to dry at room temperature and in the shade. The dried hemp leaves were ground at 28 000 *g* for 40 s and turned into a micron-sized powder. Four hundred grams (400 g) of powdered hemp leaves were transferred to a 5-litre balloon jug. Three (3) litres of distilled water was added to it. The heater was placed in the mantle and allowed to boil for 210 min after it initially boiled. The essential oils were concentrated by water cooling and the essential oil was obtained with a separating funnel. The obtained essential oils were placed in Eppendorf tubes, covered with paraffin and stored at –20 °C. Eppendorf tubes were brought to room temperature just before the surgical operation. The oil was placed in a capped tube and a 15% suspension was created with DMSO. The oil was applied to the suture area after the surgical operation. The stock tubes containing the hemp essential oil were wrapped in aluminium foil to prevent exposure to light. DMSO was used as a solvent for the essential oils. DMSO was preferred because it can pass through biological membranes and has no toxicity at low concentrations (Majno 1975; Scharffetter et al. 1989; Liska et al. 1994).

#### SUPPLY AND PREPARATION OF EXPERIMENTAL ANIMALS

The experimental animals were obtained from Erciyes University Experimental Research Application and Research Center. The rats received for experimentation were checked by the veterinarian for their health status, sex and weight. Female rats were not included in the experiment due to their menstrual cycle and hormonal balance. The weight of each rat used in the experiment ranged from 190 to 253 grams. Wistar Albino male rats were used in the study. The received rats were kept in the study room for 7 days without any treatment so that they got accustomed to it. The rats were randomly divided into 4 groups of 12 animals. During the experiment, a 12/12-hour night/day period was created and the rats were given standard rat pellet food and tap water *ad libitum*. The room temperature was maintained between 20.0 and 21.4 °C.

#### CREATION OF THE INCISION MODEL

During the night, before the working day, the rats were not given food, but they continued to drink water. All the rats were anaesthetised with 8.00 mg/kg of xylazine (Bioveta PLC, Ivanovice na Hane, Czech Republic) and 60.00 mg/kg of ketamine (Richter Pharma AG, Wels, Austria). The abdominal region was sterilised. After sterilisation, the abdominal region was shaved with a disposable razor blade. A sterile surgical drape was covered, leaving the abdominal area open. The abdomen was sterilised again and the surgical operation was started. A 4 cm long cut of the skin was performed with a scalpel. The peritoneum was opened. The peritoneum was not damaged. This area was then closed with sutures. Each layer was sutured with a simple separate suture technique. The incision was closed with 4 sutures for each rat. The suture zone was 3–3.5 cm long and 1.6 cm wide.

- Subsequently, the experimental animals were divided into four groups.
- Group 1: Povidone-iodine group.
- Group 2: Hemp seed oil group.
- Group 3: Hemp essential oil group.
- Group 4: CBD group.

After the surgical operation, the suture was closed with sterile gauze pads and the rats were placed in separate cages due to the possibility of damaging each other.

### Wound care process

The 1^st^ group, the povidone-iodine group: After the surgical operation, 10% povidone iodine was applied on the suture and on a circular area, 2 cm in diameter, around the suture area.

The 2^nd^ group, the hemp seed oil group: After the surgical operation, hemp seed oil was applied on the suture and on a circular area with a diameter of 2 cm around the suture area.

The 3^rd^ group, the hemp essential oil group: After the surgical operation, hemp essential oil was applied on the suture and on a circular area with a diameter of 2 cm around the suture area.

The 4^th^ group, the CBD group: After the surgical operation, CBD was applied on the suture and on a circular area with a diameter of 2 cm around the suture area.

After each group’s own solution was applied, the sutures of the rats were closed with sterile gauze. The rats were given one dose of antibiotics and one dose of painkillers according to their weight. Each rat was placed in a separate cage in case the rats harmed each other. The wound care product applied after the surgery was applied in the morning every day for 10 days. The cages, feed and water were changed daily due to the possibility of infection.

Evaluation of the wound tissue: The day when the incision was made was considered Day 0, and the suture area of all the rats was photographed every day. The wound was evaluated histologically on the 1^st^, 2^nd^, 3^rd^, 10^th^ and 21^st^ days. The photographs were evaluated by a histologist, independent from the study.

### Preparation of plasma

Plasma was directly collected from the heart and carefully transferred to centrifuge tubes to conduct analysis while avoiding haemolysis. It was then centrifuged at 3 000 *g* for 30 minutes. The supernatants were placed into Eppendorf tubes with transfer pipettes and were stored at –20 °C until analysis.

### Biochemical analysis

Enzyme-linked immunosorbent assay (ELISA) analyses were performed in the Erciyes University Medical Biochemistry laboratories (Kayseri, Türkiye). On the day of the analysis, the samples were removed from the –20 °C storage. In the samples taken, MCP-1, IL-8, BFGF, EGF and COL-1 were analysed by the ELISA method. The ELISA analysis was performed according to the kit’s instructions.

### Statistical analysis

The data were evaluated using the SPPS (v26; IBM, Chicago, USA) program. The descriptive data were given as numbers, percentages, the mean, standard deviation, minimum and maximum. The conformity of the data to the normal distribution was evaluated with the Shapiro-Wilk test. It was found that the data fit the normal distribution. A one way analysis of variance (ANOVA) was used in the analysis of the data. Post hoc, Bonferroni and Games-Howell tests were applied to determine which group caused the difference. The statistical significance level was accepted as *P* < 0.05.

## RESULTS

### Histological results

When the wound tissue was observed on the 1^st^ day, no signs of infection, oedema and redness were observed on the wound. For the 1^st^ day, stability was achieved in the wound tissue of the 2^nd^ and 3^rd^ group rats. Although the 1^st^ and 4^th^ groups were evaluated as good, it can be said that the wound tissue of the 2^nd^ and 3^rd^ groups was healthier.

When the wound tissue was evaluated on the 2^nd^ day, it can be stated that the wound tissue of the 3^rd^ group rats was better than the other groups, the wound tissue began to become evident, and no signs of active infection were observed in the groups.

When the wound tissue was evaluated on the 3^rd^ day, it can be stated that the wound development process of the 2^nd^ group is in a very good condition, the other groups are in similar conditions to the 2^nd^ day and there was no sign of infection.

In the evaluation of the wound tissue on the 10^th^ day, it was observed that the skin hairs were getting longer. It was not considered a distinctive feature because there were rats with or without skin hair in each group. It can be stated that the wound tissue healed completely in the Group 2 rats, there were no sutures (scars), the incision site was not visible to the naked eye, and similar findings occurred in Group 3. On day 10, the incisions in Groups 1 and 4 had not healed completely. It was observed that a 0.75 cm non-healing incision line remained from the 4 cm incision. It can be stated that there was localised tissue stiffness and stability was not provided.

In the evaluation of the wound tissue on the 21^st^ day, when the maturation phase was passed, it was observed that the incision line of the 1^st^, 2^nd^, and 3^rd^ group rats became unclear, the skin hairs increased, the wound tissue disappeared and the wound was in the maturation phase. On the other hand, although the wound tissue of the 4^th^ group rats was healed, it was thought that there was localised damage, and partial inflammation due to the wound tissue, it was thought that the wound tissue was damaged due to itching and redness as the rats were in a free state, and it can be stated that complete healing was not achieved.

### The blood tissue analyses

**Figure 1:**
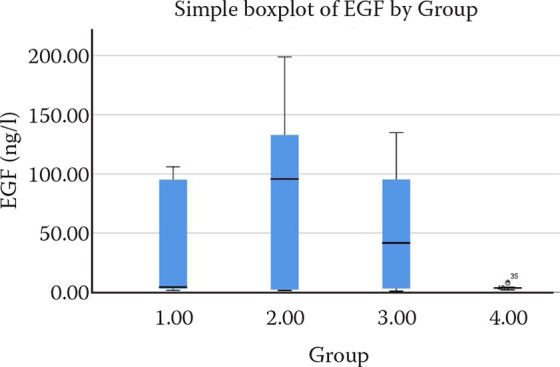
Graph of the plasma epidermal growth factor (EGF) levels

**Figure 2:**
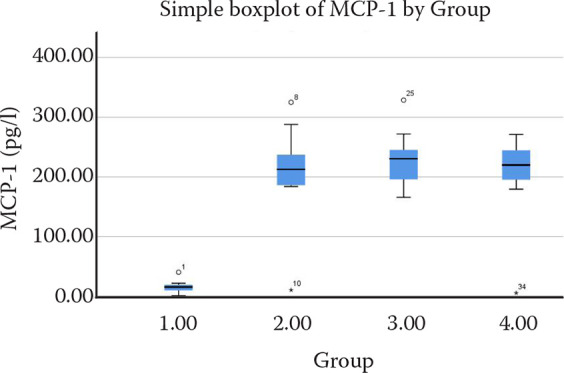
Graph of the plasma monocyte chemoattractant protein-1 (MCP-1) levels

**Figure 3:**
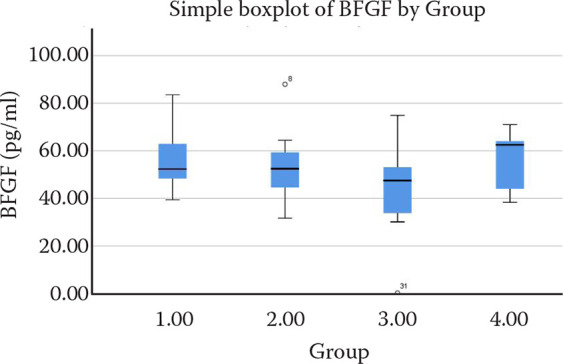
Graph of the plasma basic fibroblast growth factor (BFGF) levels

**Figure 4:**
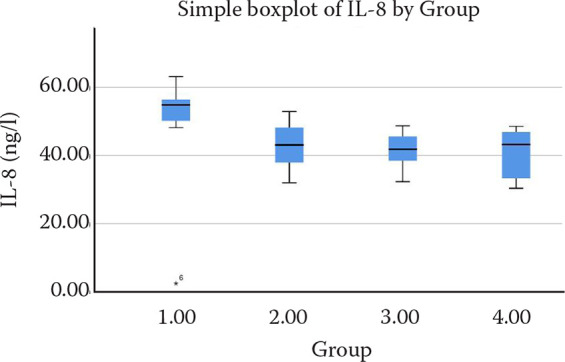
Graph of the plasma interleukin-8 (IL-8) levels

**Figure 5:**
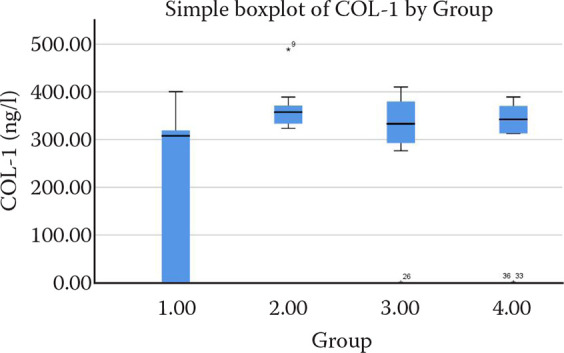
Graph of the plasma collagen-1 (COL-1) levels

The plasma EGF, MCP-1, BFGF, IL-8, COL-1 data of the rats obtained after the stages specified in the method section are given in Table 1 and Figures 1–5.

**Table 1:** Comparison of the plasma parameters between the groups

Biochemical parameters	*x̄* ± SD (min.–max.)				Test value	*P*-value
	Group 1 (a)	Group 2 (b)	Group 3 (c)	Group 4 (d)		
EGF (ng/l)	43.95 ± 51.46 (1.55–105.97)	77.37 ± 71.95 (142–198.75)	53.15 ± 54.37 (0.85–134.85)	4.29 ± 2.34 (1.88–9.14)	3.489	0.025
	b > d, c > d, a = b = c, a = d*					
						
MCP-1 (pg/ml)	16.38 ± 12.71 (1.05–40.16)	208.31 ± 75.96 (10.32–324.87)	229.12 ± 44.14 (165.66–328.58)	203.53 ± 74.89 (5.41–271.16)	20.939	0.000
	b = c = d > a*					
						
BFGF (pg/ml)	56.82 ± 14.95 (39.41–83.55)	53.13 ± 14.84 (31.67–87.87)	43.89 ± 18.35 (0.22–74.90)	56.50 ± 11.96 (38.34–71.02)	1.647	0.195
						
IL-8 (ng/l)	47.66 ± 20.46 (2.45–63.18)	43.08 ± 6.29 (32.00–52.91)	41.53 ± 5.37 (32.33–48.71)	40.89 ± 6.83 (30.40–48.56)	0.743	0.533
						
COL-1 (ng/l)	336.58 ± 43.34) (307.67–400.31)	362.83 ± 44.72 (323.45–488.92)	341.06 ± 48.24 (276.42–410.14)	353.08 ± 26.17 (312.88–389.18)	0.673	0.575

*Statistical differences

BFGF = basic fibroblast growth factor; COL-1 = collagen-1; EGF = epidermal growth factor; IL-8 = interleukin-8; MCP-1 = monocyte chemoattractant protein-1

The plasma EGF values were similar in Group 1, Group 2 and Group 3 (*P* > 0.05), the EGF levels were lower in Group 4 compared to the other groups (*P* < 0.001, *P* < 0.005), the MCP-1 values in Group 2, Group 3 and Group 4 were similar (*P* > 0.05), Group 1 had lower MCP-1 levels compared to the other groups (*P* < 0.001, *P* < 0.005), it was determined that the plasma BFGF, IL-8 and COL-1 values of the groups were similar (*P* > 0.05) (Table 1, Figures 1–5).

## DISCUSSION

In contemporary medical practices, tissue in the post-operative period remains pivotal for patients to resume their normal lives promptly (Majno 1975; Scharffetter et al. 1989; Liska et al. 1994; Konca and Akbay 2000). This study aimed to assess the efficacy of hemp essential oils, hemp seeds, and CBD in abdominal sutured incisions in rats, concurrently evaluating their effectiveness against the routine wound care agent, povidone-iodine.

Wound healing involves a myriad of biochemical parameters (Nunan et al. 2014). Firstly, we examined the epidermal growth factor (EGF).

Previous studies highlighted the positive contribution of increased EGF levels to wound healing (Busanello-Costa et al. 2023; Kim et al. 2023; Yasti et al. 2023). In our study, the plasma EGF level in Group 2 was significantly higher. Although Group 3 exhibited a higher EGF level than Group 1, the difference was not statistically significant. This suggests that the hemp seed oil may enhance the EGF levels. Notably, there is a gap in the literature regarding the impact of hemp extract on the EGF. However, a study investigating a standardised *Cannabis sativa* L. ethanolic extract containing cannabidiol demonstrated the potential effects on the vascular endothelial growth factor release (Kurtoglu et al. 2014).

Monocyte chemoattractant protein-1 (MCP-1) is another crucial parameter in wound tissue healing. Studies have reported that a decrease in the MCP-1 expression indicates that the wound tissue progresses toward healing (Aguayo-Morales et al. 2023; Chen et al. 2023; Zhang et al. 2023). In our study, it was determined that the 21^st^ day plasma MCP-1 level of the group treated with the 10% povidone iodine solution was lower than the other groups and this was statistically significant. It can be stated that povidone-iodine solution provides a lower amount of MCP-1 expression than the products obtained from hemp. In another conducted study, it was determined that THC reduces the MCP-1 secretion (Sangiovanni et al. 2019). Another study found that hemp extracts suppress MCP-1 (Busanello-Costa et al. 2023). There is also a study demonstrating that a high-CBD extract reduces the MCP-1 levels in a lung inflammation mouse model (Aguayo-Morales et al. 2023). In our study, contrary to the literature, it was found that povidone-iodine suppresses MCP-1 to a greater extent. Moreover, CBD without THC and hemp essential oils containing trace amounts of THC exhibited the same effect as the hemp seed. Therefore, it can be stated that the effect on MCP-1 is not solely attributed to THC, but also to the presence of both THC and CBD together, or solely to CBD (Aguayo-Morales et al. 2023).

Yang et al. (2014) and Ebrahimpour-Malekshah et al. (2023) showed, in their studies, that increased BFGF levels indicate the rapid healing of the wound tissue. In our study, it was determined that there was no statistically significant difference between the BFGF levels between the groups and that all the groups were similar to each other. No study evaluating the direct effects of hemp extracts on BFGF was found in the literature. However, in our study, materials containing *Cannabis sativa* showed a significant increase in cell proliferation compared to collagen hydrogels containing silver nanoparticles. Our study findings are supportive of the literature. It can be stated that CBD and hemp extracts increase BFGF at least as much as povidone-iodine (Chen et al. 2023).

Rizzo et al. (2023), Alyami et al. (2023) and Jayabal et al. (2023), in their studies, reported that the wound tissue increased the IL-8 expression. In our study, on the other hand, it was determined that there was a similar IL-8 expression without any statistical difference between the groups. None of the hemp products that we used for the experimental groups were different from the 10% povidone-iodine solution for the IL-8 levels. In a study utilising a standardised *Cannabis sativa* ethanolic extract (CSE) containing 5% CBD and a low THC concentration, it was observed that both CSE and CBD inhibited the nuclear transcription factor κB (NF-κB). However, only CSE exhibited a decrease in the interleukin-8 (IL-8) secretion. Another study reported that an extract fraction derived from the *C. sativa* Arbel strain (F CBD) significantly reduced the IL-6 and IL-8 levels in an alveolar epithelial (A549) cell line, dose-dependently (Konca and Akbay 2000; Fumagalli et al. 2022).

In our study, hemp essential oils containing CBD and low levels of THC, as well as hemp seeds, were found to suppress the IL-8 factor. These substances exhibited similar effects to each other and were comparable to the routine wound care material povidone-iodine. In the literature, no study was encountered evaluating the superiority of CBD and THC over each other or over any commonly used material (wound care product) in terms of the IL-8 factor. Therefore, it can be stated that CBD and hemp extracts suppress IL-8 at least as effectively as povidone-iodine.

Collagen contributes to the mechanical strength and flexibility of tissues and serves as a natural substrate for cellular adhesion, proliferation, and differentiation. Narisepalli et al. (2023), Hu et al. (2022) and Gong et al. (2022) reported, in their studies, that an increased COL-1 expression showed that wound healing was in a normal state.

In our study, none of the hemp products used for the experimental groups was different from the 10% povidone-iodine solution. It provided a similar COL-1 expression.

No study evaluating the direct effects of hemp products on COL-1 was found in the literature. However, in a study assessing the superiority of pure CBD over a routine care product in a wound opened on mice tongues, it was noted that the groups treated with CBD exhibited well-organised accumulated collagen fibres, newly formed blood vessels, and fibroplasia. The marginal epithelium was also reported to be thicker (Bellocchio et al. 2023).

In our study, histologically, groups treated with CBD and hemp extracts for wound care exhibited well-organised accumulated collagen fibres, newly formed blood vessels, and fibroplasia on the 10^th^ day. On the 21^st^ day, the CBD, hemp seed oil, and hemp essential oil groups showed complete wound tissue formation, while the povidone-iodine group still displayed local damage in mice. Moreover, no signs of infection were observed in the wounds. In another study, hemp extracts were indicated as a valuable source of biologically active substances that can reduce oxidative stress and positively influence the vitality of skin cells (Zagorska-Dizok et al. 2021).

Our study findings are consistent with the literature; however, it can be said that hemp seed oil and hemp essential oil are superior in terms of collagen production and wound tissue development. The reason for this could potentially be attributed to the trace amounts of THC present in these compounds.

As a result, the phytocannabinoids, terpenes and other molecules in the hemp seed oil, from the first days of the experiment, showed a good healing process that could be differentiated from the other groups. Generally, there was general well-being in the 1^st^ group, 2^nd^ group, 3^rd^ group and 4^th^ group of rats. No signs of infection were found. The recovery rate in the Group 1 and 2 rats was different from the other groups. It was determined that this may be due to the effect of THC, albeit in trace amounts. It is considered that the decrease in the stress levels facilitated the activation of the immune system and may accelerate the molecular mechanism. However, there was no drowsiness or confusion. The hemp seed oil and hemp essential oil increased the EGF at a higher rate than other groups, improving the wound healing process.

It can be said that the CBD, hemp seed oil and hemp essential oil are at least as safe as the 10% povidone-iodine in the wound healing process, and they even have superior qualities over povidone-iodine. Since CBD has no superiority over cannabis extracts in the wound healing process, the use of cannabis extracts, which are easier and more effective than pure CBD, can be recommended. It can be used in routine wound care because the solution prepared from cannabis is cheap, natural, does not cause infection, and does not put the thyroid glands at risk because it does not contain iodine.
